# M1/M2 macrophages and their overlaps – myth or reality?

**DOI:** 10.1042/CS20220531

**Published:** 2023-08-02

**Authors:** Zuzana Strizova, Iva Benesova, Robin Bartolini, Rene Novysedlak, Eva Cecrdlova, Lily Koumbas Foley, Ilja Striz

**Affiliations:** 1 Department of Immunology, Second Faculty of Medicine, Charles University and University Hospital Motol, V Uvalu 84, 15006, Prague, Czech Republic; 2 Chemokine Research Group, Institute of Infection, Immunity and Inflammation, College of Medical, Veterinary and Life Sciences, University of Glasgow, Glasgow G12 8TT, U.K.; 3 Third Department of Surgery, First Faculty of Medicine, Charles University and University Hospital Motol, V Uvalu 84, 15006, Prague, Czech Republic; 4 Department of Clinical and Transplant Immunology, Institute for Clinical and Experimental Medicine, Prague, Czech Republic; 5 Institute of Immunology and Microbiology, First Faculty of Medicine, Charles University, Prague, Czech Republic

**Keywords:** cancer, differentiation, infection, M1/M2, macrophages, polarisation

## Abstract

Macrophages represent heterogeneous cell population with important roles in defence mechanisms and in homoeostasis. Tissue macrophages from diverse anatomical locations adopt distinct activation states. M1 and M2 macrophages are two polarized forms of mononuclear phagocyte *in vitro* differentiation with distinct phenotypic patterns and functional properties, but *in vivo*, there is a wide range of different macrophage phenotypes in between depending on the microenvironment and natural signals they receive. In human infections, pathogens use different strategies to combat macrophages and these strategies include shaping the macrophage polarization towards one or another phenotype. Macrophages infiltrating the tumours can affect the patient’s prognosis. M2 macrophages have been shown to promote tumour growth, while M1 macrophages provide both tumour-promoting and anti-tumour properties. In autoimmune diseases, both prolonged M1 activation, as well as altered M2 function can contribute to their onset and activity. In human atherosclerotic lesions, macrophages expressing both M1 and M2 profiles have been detected as one of the potential factors affecting occurrence of cardiovascular diseases. In allergic inflammation, T2 cytokines drive macrophage polarization towards M2 profiles, which promote airway inflammation and remodelling. M1 macrophages in transplantations seem to contribute to acute rejection, while M2 macrophages promote the fibrosis of the graft. The view of pro-inflammatory M1 macrophages and M2 macrophages suppressing inflammation seems to be an oversimplification because these cells exploit very high level of plasticity and represent a large scale of different immunophenotypes with overlapping properties. In this respect, it would be more precise to describe macrophages as M1-like and M2-like.

## Introduction

Macrophages represent a heterogeneous and multifunctional population of differentiated mononuclear phagocytes involved in all phases of the immune response from its initiation and triggering of adaptive immunity to the resolution of inflammation and reparation of the affected tissue. Furthermore, macrophages are important also for tissue development, remodelling and homeostasis [1].

A subset of mucosal and tissue macrophages in adults originates from their precursors, peripheral blood monocytes, which are attracted in response to chemotactic stimuli. Other subsets, such as brain microglial cells and liver Kupffer’s cells, populate their compartments in early embryogenesis from the yolk sac or foetal liver and their numbers are maintained by proliferation without requirement of progenitors as some mature macrophages are able of self-renewal in response to specific stimuli [2,3]. Interestingly, macrophages can also be derived from myeloid-derived suppressor cells (MDSCs), particularly following their recruitment to the tumor site. In such cases, MDSCs often undergo a phenotypic and functional shift, acquiring macrophage-like characteristics and becoming tumor-associated macrophages (TAMs). Tissue macrophages from different organs or tissue compartments have considerably diverse specific gene expression patterns and distinct sets of transcription factors determining their properties [4,5]. They can also be broadly classified based on their location and function, as shown in Table 1 [6–23]. Furthermore, when fully differentiated macrophages from one site are transferred into different tissue, they are able to reshape gene expression profiles in response to the new environment [24].

**Table 1:** Diverse nomenclature and functions of macrophages

Tissue	Nomenclature	Predominant functions
Lung	Alveolar macrophages, interstitial macrophages	Phagocytosis and degradation of inhaled particles and microorganisms, metabolism of surfactant, regulation of mucosal proteases/anti-proteases balance [6,7]
Liver	Kupffer cells	Removal of particles, apoptotic cells, immune complexes and debris from portal circulation, phagocytosis and killing of microorganisms, recycling of hemoglobin from ingested senescent erythrocytes [8,9]
Kidney	Renal macrophages (glomerular and interstitial)	Removal of immune complexes and apoptotic cells, regulation of homeostasis and repair, phagocytosis and killing of microorganisms [10,11]
Spleen	Marginal metallophilic macrophages, marginal zone macrophages, red pulp macrophages	Phagocytosis and killing of blood microorganisms, removal of apoptotic cells, communication with T and B cells, removal of senescent erythrocytes (in red pulp) [12,13]
Brain	Microglial cells, perivascular macrophages, leptomeningeal macrophages, dura mater macrophages	Pruning synapses, phagocytosis of debris, uptake of amyloid, enhancing learning and memory, neurogenesis and remyelination, phagocytosis and killing of microorganisms [14,15]
Bone	Osteoclasts, osteal macrophages	Degradation of bone to initiate normal remodelling, regulation of bone formation and osseous healing, phagocytosis and killing of microorganisms [16,17]
Joints/cartilage	Synovial cells type A	Phagocytosis of cell debris or foreign substances in joint cavities, phagocytosis and killing of microorganisms and antigen presentation [18,19]
Connective tissue	Tissue macrophages (histiocytes)	Phagocytosis and killing of microorganisms, antigen presentation, immune regulation, tissue homeostasis and repair [20,21]
Skin	Langerhans cells (dendritic cells)	Phagocytosis and killing of microorganisms, migration to lymph nodes, antigen presentation and regulation of adaptive immune responses (activation/tolerance) [22,23]

Macrophages can be broadly classified based on their location and function, with different nomenclature used to describe their various phenotypes. The functional properties of macrophages are highly context-dependent.

The original concept of M1 and M2 macrophages based on different metabolic programs and related to previously described ‘classically’ and ‘alternatively’ activated macrophages was linked to already well accepted Th1/Th2 paradigm and became widely used not only in experimental models but also in clinical settings [25,26]. Several single-cell RNA sequencing (ScRNA seq) analyses have also been conducted in human monocytes that differentiated into M1 and M2 macrophages. These analyses identified distinct transcriptional profiles and revealed specific gene expression patterns associated with pro-inflammatory (M1) and anti-inflammatory (M2) phenotypes [27].

However, at present, this view of pro-inflammatory M1 macrophages and M2 macrophages suppressing inflammation seems to be an oversimplification because these cells exploit very high level of plasticity in response to microenvironmental stimuli and thus represent a large scale of different immunophenotypes with overlapping functional properties. In animal models, an alternative nomenclature reflecting the source of macrophages, defined stimuli and collection of markers has been already proposed [28]. On the other hand, taking into account all the limitations, studying M1-like and M2-like macrophage phenotypes and their overlaps seem to be still useful for our understanding of different immunopathological reactions and diseases and might lead to potential new therapeutical approaches.

## Mononuclear phagocyte differentiation and plasticity

Early study of van Furth and Cohen used *in vivo* labelling of mononuclear phagocytes to show that promonocyte-derived monocytes are able to migrate to peritoneal cavity to differentiate into macrophages [29]. Peripheral blood monocytes represent an important pool of tissue macrophages precursors in most tissue compartments, particularly under inflammatory conditions while other resident macrophages originate from recruited foetal monocytes and primitive macrophages in early embryogenesis and their numbers are retained by proliferation [30]. Particularly, microglia, resident CNS macrophages, originate almost exclusively from yolk sac progenitors and effectively proliferate to maintain their numbers [31].

Peripheral blood monocytes as circulating precursors of resident macrophages are heterogeneous population of cells which can be subdivided based on the expression of CD14 (LPS receptor) and CD16 (FcγRIII) [32]. The traditional monocytes called ‘classical’ are characterized by very high expression of CD14 and the absence of CD16 on their surface (CD14^++^CD16^−^) and represent prevailing population of peripheral blood monocytes. In intermediate (CD14^+/low^CD16^+^) monocytes, the expression of CD14 is substantially lower and regular CD16 membrane staining pattern can be detected. Finally, subpopulation of non-classical monocytes (CD14^+^CD16^++^) is characterized by a limited expression of CD14 and prominent CD16 membrane expression [33].

In the lung, classical monocytes represent the predominant source of interstitial and alveolar macrophages while non-classical monocytes expressing CD16 seem to differentiate into intravascular pulmonary macrophages distinct from peripheral monocytes in terms of morphology, phenotype and gene expression [34]. According to experimental data, non-classical monocytes might extravasate into inflamed tissue to differentiate into M2-like, wound healing macrophages involved in repair mechanisms [35].

Differentiation of monocytes into macrophages in response to specific signals is a complex process that involves diverse growth factors and cytokines [36–38], or contact dependent cell-cell interactions [39–41], which may vary depending on the specific tissue or organ where the mononuclear phagocytes are recruited. In this respect, macrophages from different body compartments may differ in their functional activities and also responsiveness to various signals, as shown in Table 2 [42–58].

**Table 2:** Summary of the phenotypic characteristics of M1 and M2 macrophage subpopulations in humans and mice

Phenotype	Stimuli	Regulators	Cytokines	Chemokines	Other mediators	Cell surface molecules/Markers	Function/Pathology	Ref.
M1	IFN- γ	STAT1	IFN-γ IL-1β IL-6 IL-8	CXCL9	iNOS	CD16	Pro-inflammatory	[42–53]
	LPS	IRF1	IL-10^low^ IL-12 IL-18 IL-23 TNF-α	CXCL10	ROS	CD32	Stimulation of Th1 immune response	
	TNF-α	IRF5	Type I	CXCL11		CD64	Antigen presentation	
	GM-CSF	NF-κB	interferons			CD80/86	Phagocytosis	
						HLA-DR	Tissue damage	
M2a	IL-4	STAT6	IL-10	CCL17	Arg-1	CD200R	Anti-inflammatory	[42–45,48–56]
	IL-13	IRF4	TGFβ1	CCL18	FIZZ1/RELMα (mouse)	CD163	Stimulation of Th2 immune response	
		PPARγ	IL-1RA	CCL22	YM1 (mouse)	CD206	Wound healing	
			IL-13			CD209 (DC-SIGN)	Allergy	
			IL-12^low^			CD301 (CLEC10A)		
			IL-23^low^			Dectin-1		
						CXCR1		
						CXCR2		
						TGM2		
						MHC-II^low^		
						CD11b (mouse)		
M2b	IC + TLR/IL-1R ligands	IRF3	IL-1β	CCL1	SPHK1/2	LIGHT (mouse)	Immunoregulation	[42–44,48–53]
			IL-6	CCL20		CD86	Tumour progression	
			IL-10			HLA-DR?		
			TNF-α					
M2c	IL-10	STAT3	IL-10	CXCL13	MerTK	CD163	Tissue repair/wound healing	[42–44,48–52,55,57]
	GC	SMAD2	TGF-β			CD206	Phagocytosis of apoptotic cells	
	TGF-β						Fibrosis	
M2d	Adenosine signalling		IL-10	CCL5	VEGF		Anti-inflammatory	[42–44,48–52,55,58]
	IL-6		IL-12^low^	CXCL10	iNOS (mouse)		Angiogenesis	
			TNF-α^low^	CXCL16			Tissue remodelling	
			TGF-β					

A table providing a concise overview of the distinct activation states and functional properties of M1 and M2 macrophages, highlighting key molecular markers, cytokines and effector functions associated with each phenotype. Arg-1, arginase-1; CCL, CC type chemokine ligand; CXCL, CXC type chemokine ligand; CXCR, CXC type chemokine receptor; FIZZ1, found in inflammatory zone 1; GC, glucocorticoids; IC, immune complex; IFN, interferon; IL, interleukin; iNOS, inducible nitric oxide synthase; IRF, interferon regulatory factor; LIGHT, homologous to lymphotoxin, inducible ligand, competes with herpes simplex virus (HSV) glycoprotein D for binding to HSV entry mediator, expressed on T cells; LPS, lipopolysaccharide; MerTK, Mer receptor tyrosine kinase; PPARγ, peroxisome proliferator-activated receptor-γ; ROS, reactive oxygen species; STAT, signal transducer and activator of transcription; VEGF, vascular endothelial growth factor; YM1/Chil3, chitinase-like protein 3.

M1 and M2 macrophages are two polarized forms of mononuclear phagocyte *in vitro* differentiation with distinct phenotypic patterns and functional properties, but the expression of the main polarisation markers for M1/M2 macrophages *in vitro* is highly affected not only by a specific stimulus but also the stimulation time used [59]. The situation is even more complicated *in vivo*; there is obviously a wide range of different macrophage phenotypes in between depending on the microenvironment and natural signals they receive.

Polarized M1 macrophages are effector cells induced by pro-inflammatory cytokines, such as tumour necrosis factor α (TNF-α) and interferon-γ (IFN-γ) or by other pro-inflammatory stimuli including bacterial lipopolysaccharide (LPS), [60,61]. Their essential role in eliciting antibody-dependent cellular phagocytosis (ADCP) is undisputable in pathologic conditions, such as cancer and infection [62].

Differentiation into M1 macrophages is dependent also on growth factors: granulocyte-macrophage colony-stimulating factor (GM-CSF) and macrophage colony-stimulating factor (M-CSF) [63]. M1 macrophages are able to release high levels of pro-inflammatory cytokines IL-1β, IL-6, TNF-α [64,65] and IFNγ [66], and may induce Th1 differentiation by IL-12 secretion [67]. High levels of inducible nitric oxide synthase (iNOS) induce nitric oxide (NO) and together with reactive oxygen species (ROS) mediate potent killing activity of intracellular pathogens [68].

M2 macrophages produce high levels of anti-inflammatory cytokines, such as IL-10 and TGF-β [69]. They are involved in tissue repair and remodelling, and play an important role in suppressing the immune response. There are several subsets of M2 macrophages that have been identified according to their specific functions and surface marker expression. On the other hand, it’s important to note that these subsets of M2 macrophages are not entirely distinct from each other and overlaps may exist in terms of phenotypic patterns and functional activities.

M2a macrophages are induced by IL-4 and IL-13 and regulate tissue repair and wound healing [70]. They express high levels of the mannose receptor (CD206) allowing them to phagocytose apoptotic cells and debris and healing mechanisms [71].

M2b macrophages are induced in response to immune complexes and Toll-like receptor (TLR) agonists including LPS [72] and produce high levels of anti-inflammatory IL-10 and CCL1 [73]. M2b macrophages expressing high levels of CD86 [74] may play a role also in antigen presentation.

M2c macrophages are differentiated by IL-10 and glucocorticoids [75], and are involved in late phases of immune reactions and in resolution of inflammation. M2c macrophages are characterized by high expression of the scavenger receptor CD163, which plays a role in the clearance of haptoglobin/hemoglobin complexes [76,77].

M2d macrophages undergo their differentiation by adenosine receptor agonists stimulation independently on IL-4Rα signalling [78] and are associated not only with tissue repair [79] but also with *in vitro* cancer cell invasion [80].

**Figure 1:**
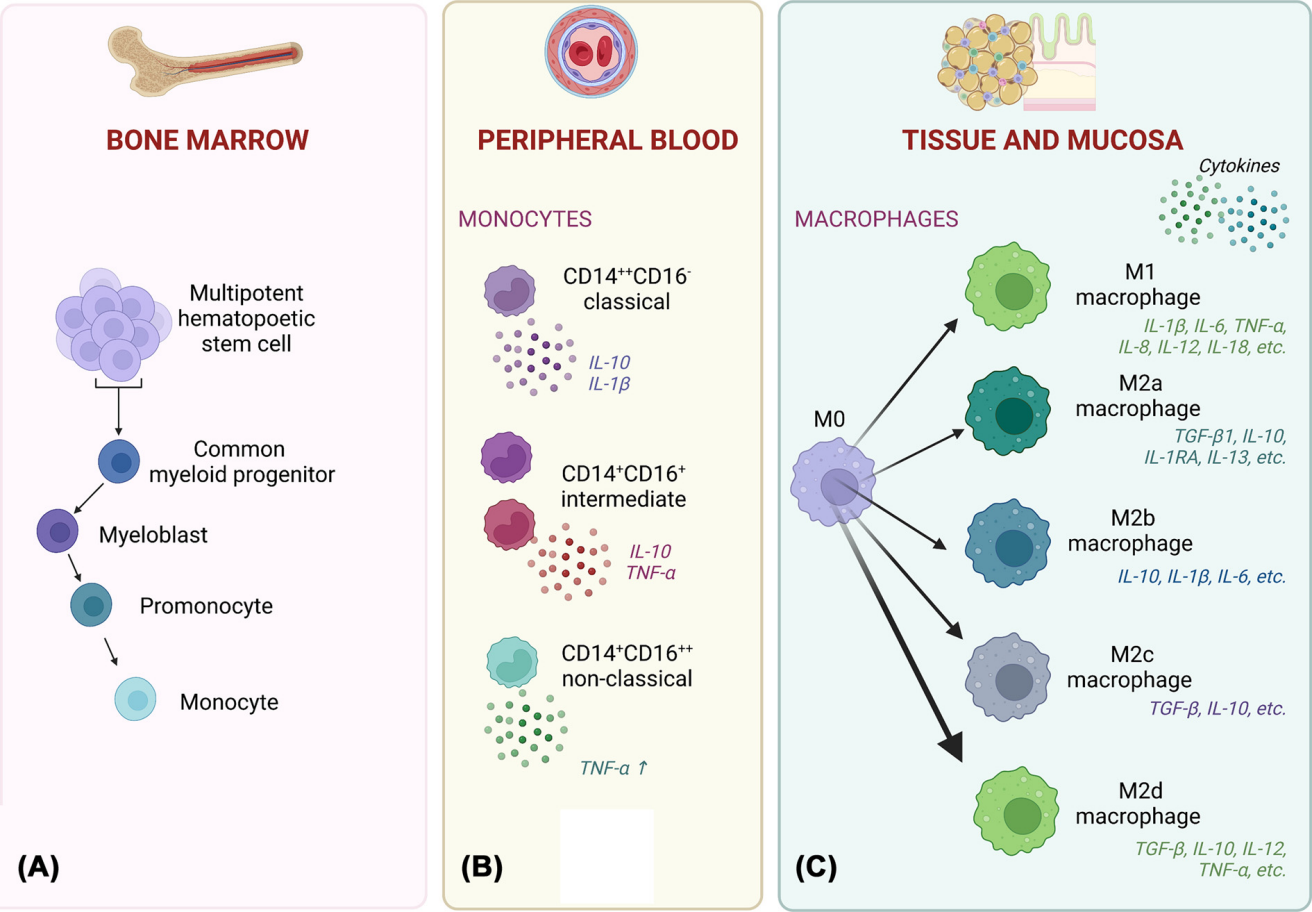
Monocyte differentiation and macrophage polarization (**A**) Multipotent hematopoietic stem cells (HSCs), which reside in the bone marrow, give rise to the lymphoid and myeloid lineages. Common myeloid progenitor further differentiates into mature cell types. The steps involved in monopoiesis are myeloblasts to promonocytes to mature monocytes. (**B**) Three phenotypically and functionally heterogenic types of human monocytes are now described within the monocyte subsets in the peripheral blood. In response to different stimuli, these monocyte subsets secrete different cytokines. (**C**) In human tissues and mucosa, M0 macrophages are polarized towards M1, M2a, M2b, M2c and M2d phenotypes, each characterized by unique cytokine profiles and exhibiting specialized functions that are critical for the proper physiological and pathological processes of the organism. Created with BioRender (Ref.No.DF256UM31V). CD, cluster of differentiation; IL, interleukin; TGF, tumour growth factor; TNF, tumour necrosis factor.

It’s important to note once more that the M1/M2 classification is somewhat of a simplification, and macrophages can display overlapped phenotypes depending on their tissue location and type of signalling. Several hybrid phenotypes have been already showed in Boolean models and may play an important role, e.g., in response to tumours when M1M2d seems to be the most effective combination to fight cancer cells without excessive tissue damage while combinations M2bM2d, M2aM2d, and M2cM2d may lead to tumour progression, M2aM2d being the worst of them [81]. As shown in Figure 1A, monocytes originate from the hematopoietic stem cell and process through various intermediate stages of differentiation. These stages include common myeloid progenitor, myeloblast, promonocyte, and finally the mature monocytes that are released into the bloodstream. Based on specific surface markers, different subsets of monocytes are commonly described (Figure 1B). Each subset has distinct functions and roles in immune responses and disease pathophysiology. Both monocytes and macrophages are characterized by unique cytokine profiles (Figure 1B,C). In consideration of the anatomical localization of macrophages, it can be inferred that they exhibit distinctive functional characteristics that are specifically adapted to suit the unique microenvironment of their respective tissue locations, as shown in Figure 1C.

## M1/M2 macrophages in infection

**Figure 2:**
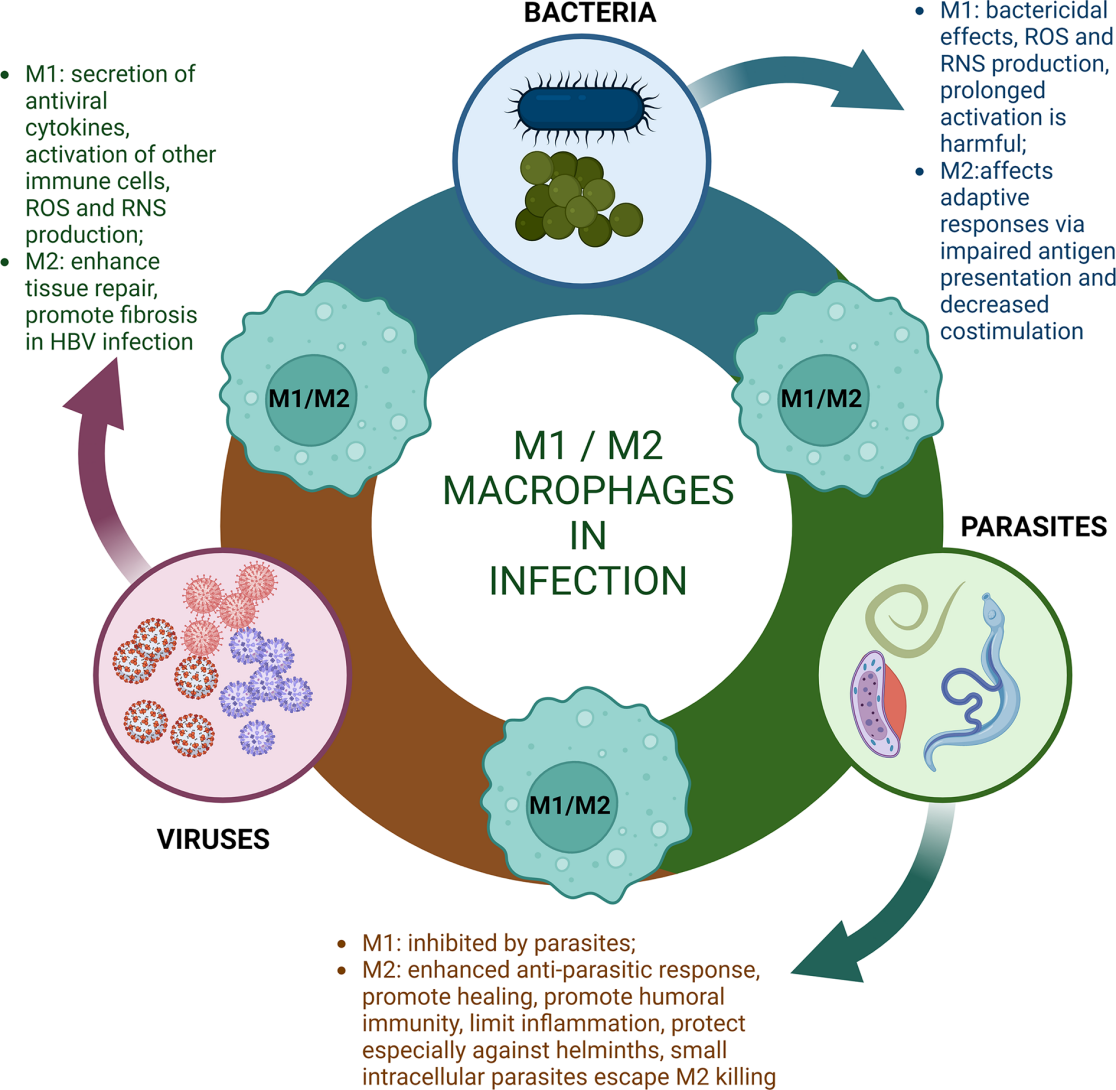
Schematic illustration of diverse roles of macrophages in infection Macrophages exhibit phenotypic plasticity and can adopt distinct activation states in response to bacteria, parasites, and viruses. Different macrophage phenotypes play diverse roles in infections ranging from bactericidal effects and cytokine secretion to tissue repair and fibrosis promotion. Created with BioRender (Ref.No.RX256VW1N3). HBV, Hepatitis B virus; RNS, reactive nitrogen species; ROS, reactive oxygen species.

*In vivo* studies of macrophage polarization during infection have been confounded by the often-opposing signals pathogens and the host release to modulate macrophage behaviour towards an advantageous phenotype. Indeed, pathogens generally tend to prefer and release mediators to skew macrophage polarization towards an anti-inflammatory phenotype, which is permissive to infection [82], while the host will attempt to enhance the bactericidal properties of macrophages by favouring M1 polarization (Figure 2). This ‘tug-of-war’ of macrophage polarization between host and pathogen during infections has made it hard to underpin the exact mechanisms that regulate macrophage polarization, often resulting in the formation of intermediate M1-M2 macrophages unique to certain diseased states [83–85]. Furthermore, diverse pathogens, sometimes even different strains of the same species, are capable of inducing very different macrophage phenotypes, suggesting macrophage polarization during infection needs to be studied in a case-dependent way [86].

### Viruses

M1 polarized macrophages play essential roles in fighting against viral infections through multiple strategies, including for the production of an oxidized environment via the secretion of ROS (reactive oxygen species) and RNS (reactive nitrogen species), antiviral cytokines and by activating other immune cells [87].

Directly after virus recognition, macrophages endocytose the invading pathogens, polarize to the M1-like phenotype [88] and present viral peptides via the major histocompatibility complex (MHC) to T lymphocytes, which in turn produce IFN-γ and other molecules to recruit effector immune cells and induce an inflammatory response capable of controlling viral replication. Antiviral cytokines released by M1 macrophages include TNFα, IL-1, IL-6, IL-8 and IL-12, which exert antiviral activities both directly and indirectly. For example, TNFα inhibits replication of viruses such as herpes simplex, swine fever virus and influenza [89], HIV-1, [90] and hepatitis B [91], which are also capable of enhancing the cytotoxicity of other leukocytes, such as NK cells, in an IL-β, IFN-β or IL-15-dependent fashion [92].

As an M1 macrophage profile is usually deleterious to the invading virus [93,94], it is unsurprising that viruses have evolved multiple strategies to evade the anti-viral responses of M1-polarized macrophages. To achieve M2 polarization, herpesviruses (and poxviruses) secrete viral IL-10 (vIL-10) and promotes macrophage polarization to M2 via transcription factor called signal transducer and activator of transcription 3 (STAT3), [95]. Activation of STAT3 via IL-10 stimulation is also used by hepatitis C virus encoding E2 protein, which up-regulates IL-10 production and M2 polarisation [96].

While M2 polarization is supposed to enhance tissue repair, during chronic infections, M2-like macrophages can promote tissue fibrosis, neoplasia and impair Th1 responses, thus promoting pathogen persistence and associated tissue pathology [97]. This is the case with hepatitis B-virus (HBV), where HBV encoded proteins directly promote M2-like macrophage activation, resulting in uncontrolled fibrosis. Indeed, induction of M2-like macrophage in the liver is associated with accelerated liver fibrosis and necrosis in patients with acute HBV-induced liver failure [98].

### Bacteria

The common response of macrophages to bacterial infections mainly involves the up-regulation of genes involved in M1 polarization. These include genes encoding cytokines such as TNFα, IL-6, IL-12, IL-1β, cytokine receptors such as IL-7R and IL-15RA, chemokines such as CCL2, CCL5 and CXCL8, enhanced microbicidal activity and the up-regulation of co-stimulatory molecules, CD80 and CD86 [99].

Thus, similar to viral infections, M1 macrophages are usually associated with protection during acute bacterial infectious diseases. Macrophages exposed to *Listeria monocytogenes*, which causes disease in immunocompromised patients and pregnant women, polarize towards an M1 program [100], with increased expression of reactive oxygen intermediates (ROIs) and reactive nitrogen intermediates (RNIs) that prevents the escape of *L. monocytogenes* from the phagosomal vacuoles that contain them, killing the bacteria both *in vitro* and *in vivo* [101].

Detection of *Salmonella* by macrophages is capable of inducing M1 polarization in both murine and humans via TLR4 engagement [102], and this shift to M1 supports resistance to the intracellular bacteria and controls the acute phase of infection [103].

While it is clear that an M1 profile is generally protective against bacteria, an excessive or prolonged M1 program can actually be deleterious for the host [104]. *Helicobacter pylori*, the causative agent of ulcer disease and some other gastric cancers, induces an enhanced M1-like phenotype in gastric macrophages of patients with atrophic gastritis [105], a pathology characterized by chronic inflammation and increased chance of developing gastric precancerous lesions [106].

As M1 polarization tends to be bactericidal, just like in viruses, it is unsurprising that some bacteria have evolved different strategies to block M1 polarization. Mycobacteria are capable of interfering with M1 polarization through the secretion of ESAT-6, which directly inhibits the activation of M1-like transcription factors NF-κB and IFN-regulatory factors [107], preventing the formation of a granuloma and inhibiting macrophage bactericidal activity. Similarly, *Salmonella typhimurium* SP1-2 encodes for mediators that inhibit the oxidative microbicidal activity of macrophages [108]. TcpC, a virulence factor of *Escherichia coli*, has been shown to inhibit M1 macrophages in vitro and promote M2 macrophage polarisation, even if LPS and IFN-y (M1 inducers) are supplied to the cultures [109]. *Coxiella burnetii*-infected macrophages release the M2-associated molecules IL-10, TGFβ1, and CCL18 and fail to produce nitric oxide. Furthermore, co-stimulatory molecule CD80 and lymph node homing chemokine CCR7 expression are also reduced, affecting the adaptive response to the bacteria in the draining lymph node [110].

### Parasites

Unlike viruses and bacteria, parasites’ complex life cycle, multicellularity and extensive immune evasion capabilities pose unique challenges to the immune system. This is especially true for macrophages, as phagocytosis and destruction of the invader are usually not possible due to its size [111]. In these cases, the function of macrophages is to limit tissue damage as the parasite migrates through the hosts’ body and destroys cells and tissues in their path [112]. Accordingly, an M2 polarization profile focused on dampening inflammation and promoting healing is the most appropriate response against large extracellular parasites like helminths. However, the opposite is true if the parasite is small and intracellular, such as protozoa like *Leishmania* or *Toxoplasma*, as an M1 profile focused on high levels of reactive oxygen and nitrogen species that impair parasite replication and infectivity.

For example, patients infected with cutaneous Leishmaniosis show high plasma levels of arginase, TGF-β and PGE_2_ [113], suggesting the parasite is capable of promoting M2 macrophage polarization to escape killing. Indeed, *in vitro* studies have shown that only M2 macrophages allow growth of *Leishmania major* and *L. amazonensis* [114]. Similarly, *Trypanosoma brucei*’s metabolite infolepyruvate can inhibit M1 polarization and decrease IL-1β production by macrophages, contributing to immune evasion in both murine [115] and human macrophages [116].

On the other hand, during helminth infections, the host wants to maintain tissue homeostasis, promote healing and mitigate tissue damage associated with the trafficking of these large multicellular parasites through host tissues [117], while the parasites prefer an anti-inflammatory and tolerogenic environment to grow and reproduce [118].

Even though M2 polarization eventually leads to the releases of mediators that enhance an anti-parasitic response through the induction and development of the humoral immunity [119], most helminth secreted factors do not appear to push macrophage polarization towards the opposite M1 profile [120]. This is probably because helminths secrete a wide variety of immunomodulatory substances that are capable of down-regulating parasite-specific effector responses (T-cell hypo-responsiveness) [121] and affect antigen presentation [122] and B-cell antibody production [123]. In other words, helminths modulate the downstream effects of M2 macrophage polarization on the immune system without inhibiting M2 polarization directly, preserving the macrophages’ anti-inflammatory and tolerogenic properties.

While this balance creates a chronic state of infection and delays parasite expulsion, death by helminth infection is rare (even though estimates suggest that 2 billion people are currently infected with helminths) [124], and most of the burden of disease is related to chronic changes in hosts’ health and nutritional status, such as intestinal blood loss leading to iron deficiency and protein malnutrition.

While the classic M1-M2 polarization dichotomy might be too simplistic, especially *in vivo* where multiple signals from both the host and the pathogen create a unique environment, which results in the intermediate or incomplete polarization, it is also clear that in most cases both pathogen and host know exactly which type of macrophage polarization is best suited for their survival.

## M1 and M2 macrophages in anti-tumor immunity

Macrophages are present at varying frequencies in all types of cancer and account for one of the most frequent immune cells in the tumor microenvironment (TME). The precursors of the macrophage pool in the TME are primarily tissue-resident macrophages and blood monocytes, with a smaller fraction deriving from MDSCs that are closely related to blood monocytes [125–127]. While the rapid differentiation of MDSCs into macrophages within the TME may occur, it is crucial to emphasize that this process is characterized by a high degree of complexity and remains context dependent. In this scenario, the differentiation of MDSCs into TAMs shows substantial heterogeneity, both across different cancer types and among individuals, and to date studies are still investigating the factors that induce changes in gene expression and cellular signalling pathways, leading to the transformation of MDSCs into TAMs [128].

**Figure 3:**
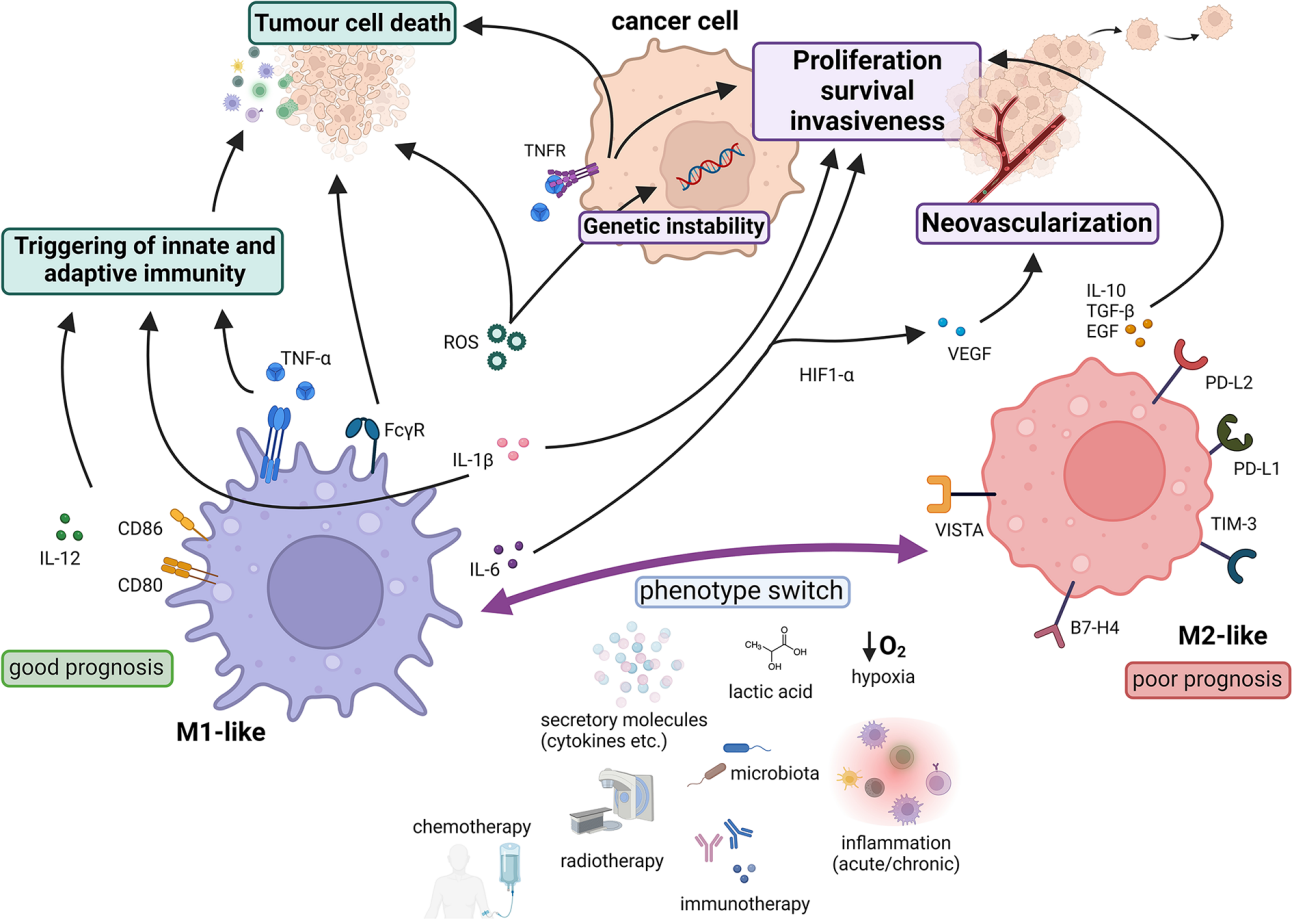
Dual role of macrophages in cancer Macrophages can exhibit distinct phenotypes with diverse functional properties that are shaped by various stimuli. M1-like macrophages are characterized by effective anti-tumor mechanisms, such as the triggering of other immune cells, phagocytosis and cytotoxicity. However, some of their molecules can paradoxically promote tumor growth, proliferation, survival, metastases formation and neovascularization, which are more commonly associated with M2-like macrophages. Despite these complexities, the presence of M1-like macrophages in tumors has generally been associated with favourable prognosis. Created with BioRender (Ref. No. SN256VZ1EJ). CD, cluster of differentiation; EGF, endothelial growth factor; FcγR, Fc γ receptor; HIF1-a, hypoxia-inducible factor 1-α; IL, interleukin; ROS, reactive oxygen species; TGF-b, tumour growth factor β; TIM-3, T-cell immunoglobulin and mucin domain-containing protein 3; TNFR, tumour necrosis factor receptor; VEGF, vascular endothelial growth factor; VISTA, V-domain immunoglobulin suppressor of T-cell activation; PD-L1/2, programmed death-ligand 1/2.

Macrophages act as double-edged swords in cancer because of their ability to provide successful anti-tumour immunity and orchestrate the immunosuppressive TME (Figure 3). Potent anti-tumour mechanisms include activation of innate and adaptive immunity, antibody-dependent cellular cytotoxicity, antibody-dependent cellular phagocytosis and direct cytotoxicity [129,130]. All these properties are attributed to properly functioning M1-like macrophages. Nevertheless, under the influence of various factors, such as local hypoxia, high levels of lactic acid, inflammation and secretory molecules (cytokines, chemokines, etc.), macrophages gain impaired functions and protumoural properties [131,132]. M2-like macrophages attempt to repair cancer tissue through immunosuppression, tissue remodeling and neovascularization. Tumor cells have learned to take advantage of these reparative mechanisms, which they find highly advantageous. Consequently, they have adapted to exploit these mechanisms to the fullest extent which bolsters the growth and development of the tumor even further [133]. M2-like macrophages express metalloproteinase, matrix remodelling enzymes and other growth factors, such as EGF, TGF-β and VEGF, which facilitate cancer cell proliferation, invasiveness, epithelial–mesenchymal transition and metastasis formation [134–136]. In addition to the production of immunosuppressive cytokines, various immune checkpoint molecules expressed by macrophages, such as B7-H4, PD-L1, PD-L2, TIM-3 and VISTA, drive immunosuppression within the TME [131,137,138].

There is no unique nomenclature system for macrophages present in the TME. Most studies consider all macrophages in the TME as tumour-associated macrophages (TAMs), which are further divided into M1/M1-like and M2/M2-like macrophages [130,132,139–142]. Some studies have described TAMs as a unique group that does not exist under normal physiological conditions and possesses a specific phenotype with both M1 and M2 characteristics [132,143,144]. Furthermore, few studies have described only M2/M2-like macrophages as TAMs [145,146]. Additionally, many researchers have distinguished between M1/M2 macrophages and M1/M2-like macrophages. M1 refers to macrophages induced in response to IFN-γ and bacterial products, whereas M1-like is a polarization state that promotes antitumour immunity and cytotoxicity. In contrast, M2 differentiation is driven by IL-4 and IL-13 while M2-like macrophages suppress effective adaptive immunity to promote protumoural conditions [125,144]. ScRNA-seq has emerged as a crucial technique for studying TAMs, enabling a deeper understanding of their plasticity and facilitating the identification of rare TAM populations. Remarkably, the exploration of these newly discovered macrophage subsets in tumors, such as CD74^+^ macrophages or macrophages with high expression of CCL8, has shed light on their potential roles in tumor progression [147].

Tumour-promoting macrophages are highly variable. Undoubtedly M2-like macrophages contribute to various hallmarks of cancer, however, one of the hallmarks is tumour-promoting inflammation, which in multiple ways may support the cancer cells [148]. M1-like macrophages largely contribute to the production of pro-inflammatory molecules such as IL-1β, IL-6, and TNF-α.

The effects of TNF-α depend on its concentration, form (soluble/membrane-bound), receptor binding, and subsequent cell signalling. TNF-α may induce apoptosis in cancer cells; however, it can also stimulate tumour growth, survival, proliferation, invasion, and metastasis formation [149,150]. Similarly, IL-6 may facilitate tumour cell survival and migration, induce cancer stemness, and prime macrophages towards the M2-like phenotype [132,151–153]. Additionally, IL-6 triggers the expression of key angiogenic key factors HIF-1α and VEGF [151]. Similarly, IL-1β has been shown to increase angiogenesis, tumour growth, and invasiveness [154,155]. Another factor that contributes to the development of tumour-promoting inflammation is ROS. Increased ROS production leads to genetic instability, another hallmark of cancer [129,156]. In conclusion, macrophages have a heterogeneous spectrum of phenotypes with M2-like macrophages being narrowly associated with tumour-promoting properties. It is worth mentioning that the M1-like macrophages can also exert these effects by facilitating the growth of cancer cells, promoting neovascularization, and contributing to the development of metastases.

TME is extremely complicated, dynamic, and quickly evolving milieu [157,158]. However, differences exist among macrophage subpopulations within a tumour, reflecting TME heterogeneity [129,131]. Besides TME, which is specific for a given tumour type and stage, the affected organ, macrophage origin, and microbiota also influence the final phenotype of macrophages. Thus, the phenotype of macrophages within the TME is complex, overlapping and plastic [129,140,159]. The classification of M1-like and M2-like macrophages is despite the complexities and numerous phenotypes of macrophages, useful for assessing the prognosis.

Data from almost 300 studies of several human cancers, including more than 70,000 patients showed conflicting effects of macrophages on patient prognosis since earlier studies failed to distinguish between M1 and M2 macrophages. However, following macrophage classification according to their phenotype, it can be concluded that M1-like macrophages positively influence patient prognosis while M2-like macrophages have the opposite effect in most cancers [145]. Several meta-analyses have confirmed these results in various cancers [160–162]. Although there are several different phenotypes of macrophages, it is clear that this classification is useful for predicting prognosis and facilitates the comparison of results from various research teams.

### Therapeutic modulation of the M1/M2 phenotype

The macrophage phenotype can be modulated using drugs and small molecules. Chemotherapeutic drugs have dual effects on the macrophages. Some drugs, such as paclitaxel may increase the number of M2-like macrophages, which try to repair the tissue and thereby support the tumour progression through mechanisms described above. Conversely, other drugs, such as trabectedin, reduce the number of macrophages in the TME, thus limiting their pro-tumoural effects. Other chemotherapeutic agents (e.g., doxorubicin) may increase tumour immunogenicity and simultaneously induces M1-like macrophages [125,163]. Alternatively, M2-like macrophages may support the chemoresistance of tumour cells through various interactions between macrophages and tumour cells, and other immune cells [163]. Macrophages are amongst the most radioresistant cells, and radiotherapy increases macrophage number within the TME. In addition, irradiation may dose-dependently influence the macrophage phenotype. Several ongoing clinical trials have combined radiotherapy with drugs that modulate macrophage functions to boost antitumour immunity [164,165]. These studies suggest that treatment modalities can also influence the final phenotype of macrophages.

Immunotherapy has immense potential for treating cancer. Therapeutic approaches targeting macrophages are mainly aimed to suppress the pro-tumoural properties of M2-like macrophages, facilitate the development and support of M1-like macrophages, and convert the macrophages from M2-like to M1-like stage. Activation of the M1-like macrophages or reprogramming of the M2-like macrophages can be achieved using monoclonal antibodies (CD40, MARCO, PD-L1, etc.), Toll-like receptor agonists, chemical compounds (GM-CSF, IFN-γ, vadimezan, etc.), miRNA, and histone deacetylase inhibitors [131,164,166–168]. The properties of M2-like macrophages can be modulated using immune checkpoint inhibitors (anti-LILRB1, anti-PD-L1, anti-VISTA, etc.) and small molecule inhibitors (against IDO, PI3K-γ, etc.) [166]. There are several approaches for targeting macrophages within the TME, some of which have already been approved, while others are being investigated in clinical trials and pre-clinical testing. Since M2-like macrophages outnumber M1-like macrophages within the TME, the therapeutic approaches aimed at macrophage reprogramming are of particular interest.

In summary, the distinction between protumoural and antitumour macrophages is not so clear, therefore, macrophages cannot be defined using the simple M1/M2 classification system. Nevertheless, this classification system can help in understanding the properties of macrophages within the TME and their effects on tumour cells. Importantly, the M1/M2 division is also associated with patients´ prognosis across different cancer types. Current treatment modalities seem to influence macrophage abundance and phenotype; however, further studies are needed to comprehensively decipher the effects of chemotherapy and radiotherapy on macrophages. Furthermore, novel immunotherapeutic strategies need to be further tested and validated to effectively reprogram M2-like macrophages and limit their protumoural functions. Nonetheless, macrophages appear to be a promising target in cancer therapy.

## M1/M2 macrophages in autoimmunity

Even though the over‐simplified description of macrophage heterogeneity based on the M1/M2 polarization may not fully express the complex role of macrophages in autoimmunity and inflammation, the pathogenic role of macrophages in autoreactive processes has been widely studied [169,170]. Since the macrophage polarization is dependent on a variety of stimuli, the inflammation scenarios may differ in the disease remission and disease flare [171]. In autoimmunity, the altered balance of M1/M2 macrophage phenotypes is considered potentially harmful as both prolonged activation of M1 macrophages, as well as altered function of anti-inflammatory M2 macrophages can trigger and further promote inflammation [171]. In addition, studies have demonstrated that progesterone and oestrogen can promote the activation of M2 macrophages and this phenomenon has been suggested as a possible contributing factor to the increased prevalence of autoimmune diseases observed in women [172,173].

Macrophages play an important role in the pathogenesis of autoimmune disorders, such as autoimmune diabetes, rheumatoid arthritis, Sjögren’s syndrome, systemic sclerosis, and systemic lupus erythematosus (SLE). Thus, their contribution to the inflammatory process has been studied in both mice and humans. Furthermore, macrophage activation syndrome (MAS) is a well-known phenomenon that has been described in a variety of autoimmune diseases [174].

### Type I diabetes mellitus

In autoimmune type I diabetes (TID), macrophages are considered the main source of pro-inflammatory cytokines in adipose tissue [175,176]. This was shown to impair the glucose metabolism and insulin sensitivity in mice. Moreover, the overexpression of TGF-β in macrophages leading to excessive fibrosis has also been associated with autoimmune diabetes [177].

Interestingly, macrophages infiltrating the pancreatic islets were shown to closely cooperate with pancreatic β cells and regulate insulin secretion by detecting endogenous ATP that is co-released with insulin [178]. Furthermore, macrophages may be the primary source of IL-1β in the islet microenvironment which is particularly relevant since pancreatic β cells were shown to have very high expression of the signalling IL-1 receptor 1 (IL-1R1) [179]. The specific mechanism by which macrophages produce IL-1β in the islet microenvironment is not fully understood, however, the macrophage-mediated secretion of IL-1β has been linked to the impairment and destruction of insulin-producing β-cells. The IL-1β also stimulates the expression of other pro-inflammatory cytokines and chemokines, as well as adhesion molecules that promote the recruitment of additional immune cells to the site of inflammation. During chronic inflammation, the pro-inflammatory cytokines further disrupt the insulin signalling leading to insulin resistance [180].

### Systemic lupus erythematosus

In systemic lupus erythematosus, both M1-like and M2-like macrophages were shown to be partially responsible for the pathogenesis of lupus nephritis [181]. While M2 macrophages seem to be the dominant phenotype in SLE, M1 macrophages presumably accompany the disease flare [181,182]. M2 macrophages are traditionally activated by anti-inflammatory signals and may lead to the resolution of inflammation. Nevertheless, in SLE, M2 macrophages may be activated in an abnormal way, leading to the production of pro-inflammatory cytokines and thus contributing to the chronic inflammation and tissue damage [183]. In addition, cyclophosphamide- treated mice with disease remission were demonstrated to have a higher load of M2 macrophages [184]. Interferon regulatory factor 5 (IRF5) hyperactivation was previously associated with onset of SLE and for that reason, was implicated as a therapeutic target [185]. Interestingly, IRF5 contributes to the macrophage polarization towards M1 phenotype [186]. Thus, it seems that M1/2 macrophage imbalance correlates with disease activity in SLE [171]. Several studies also indicated that inhibiting the activity of M2 macrophages can reduce inflammation and improve symptoms in animal models of SLE [181].

### Rheumatoid arthritis

M1 macrophages also seem to largely affect the progression of rheumatoid arthritis (RA) [187]. In RA, the synovial tissue is the defined by the presence of various pro-inflammatory cytokines, such as TNF-α, IL-1, IL-6, IL-12, and IL-23, thus creating an inflammatory microenvironment causing the damage of the joints [188]. This phenomenon also leads to the polarization of T cells and activation of fibroblasts which secrete receptor activator of nuclear factor kappa-B (NFκB) ligand (RANKL) and macrophage colony-stimulating factor 1 (M-CSF) [189]. Of note, not only the synovial macrophages but also the regulation of the signalling pathways and activation status of NFκB significantly differs in RA patients as compared with healthy individuals [19,190]. The skewed M1/M2 ratio seems to also largely affect the osteoclasts which participate in the formation of bone erosions [191]. M1/M2 ratio was shown to be the only significant factor affecting the proportions of osteoclasts [192]. Given the secretion of TGF-β and IL-10, M2 macrophages were proposed as critical players in the regression of RA inflammation. Thus, factors contributing to M2 polarization are currently widely studied for the development of novel treatments [193].

### Systemic sclerosis

As opposed to RA, in systemic sclerosis, the fibrotic nature of this disease is generally being discussed mainly in the context of pro-fibrotic profile of M2 macrophages [194]. As such, M2 macrophages are potentially the major source of fibrosis-inducing cytokines and this may in ultimately cause the skin malfunction in systemic sclerosis [195]. The complex role of TGF-β in fibrogenesis has been previously elucidated and its capacity to increase collagen synthesis, as well as the myofibroblasts differentiation, migration and adhesion seems to represent the hallmark of fibrosis [196]. Further analyses including sequencing of the transcriptome from skin biopsies have allowed the identification of M2 macrophage-associated remodelling of the extracellular matrix in patients with systemic sclerosis [195].

### Sjögren’s syndrome

Only little is known about the pathogenetic role of macrophages in the Sjogren’s syndrome. It was shown that macrophages together with CD4 T cells contribute to the destruction of oral and ocular mucosa in Sjögren’s syndrome. Furthermore, human studies, especially utilizing salivary gland biopsies, demonstrate the infiltration of macrophages and its correlation with disease severity [197]. Keratoconjunctivitis sicca, xerophthalmia, and peripheral neuropathy, among others, have been reproduced in mice. In the glandular infiltrates of these mice, a heterogeneous population of immune cells has been observed, comprising CD4+, CD8+ and B-lymphocytes, DCs, as well as macrophage/monocytes. Macrophages/monocytes of these infiltrates highly express pro-inflammatory cytokines such as IL-1β and IFN-γ in the ocular tissue [198].

Currently, more research is needed to understand the role of macrophages in autoimmunity. Additionally, it needs to be determined whether targeting these cells could bring potential benefits.

## M1/M2 macrophages in atherosclerosis

The inflammatory environment in atherosclerotic lesions seems to be important for their generation and decreased stability leading to thrombosis. Vascular macrophages regulate both the progression or regression of atherosclerosis by cell–cell interactions with other cells, affecting the cholesterol metabolism and impairing the plaque stability by modulation of matrix metalloproteinase/extracellular matrix ratio [199]. M1/M2 polarization together with metabolic reprogramming is being one of the potential factors affecting occurrence of atherosclerosis and cardiovascular diseases [200,201]. When comparing two distinct anatomical locations, macrophages with the M1 expression profile are more frequent within the carotid plaques as compared to the femoral plaques [202]. Interestingly, in symptomatic patients with acute ischemic attack, M1 macrophages were shown to predominate in the atherosclerotic plaques, while only limited number of M2 macrophages was present in those cases [203]. Nevertheless, these findings were in contrast with the asymptomatic plaques.

In human atherosclerotic lesions, macrophages expressing both M1 and M2 phenotypic markers and transcriptional profiles have been detected with M1 macrophages prevailing in rupture-prone areas potentially affecting plaque stability [204]. Also in a recent study, the presence of M1 macrophages in perivascular adipose tissue surrounding coronary arteries correlated with plaque progression, higher collagen content and destabilization with higher risk of thrombosis. However, in this study, M2 macrophages were also associated with atherosclerosis by increased plaque size, calcification and higher necrotic content [205]. Furthermore, the presence of M2 related CD163+ macrophages and CD163 mRNA in human carotid plaques was found to be associated with increased plaque vulnerability [206]. These findings are in contrast with previous studies showing CD163+ macrophages as atheroprotective cells [207,208]. In this respect, M1/M2 concept of pro-atherogenic M1 macrophages and atheroprotective M2 macrophages doesn’t look to be fully applicable. Similarly, meta-data analysis of public micro-array datasets of M1/M2 genes comparing early and advanced human atherosclerotic plaques did not prove clear polarization of macrophages [209].

Macrophages in adipose tissue may be also polarized, such as in epicardial adipose tissue of patients with coronary artery disease, the M1/M2 ratio is increased as compared to a control group and correlates with the disease severity [210].

## M1/2 macrophages in allergy

Mononuclear phagocytes play a crucial role also in allergic diseases and polarization of these cells have been studied particularly in bronchial asthma. Although the immunopathogenesis of allergic asthma is focused mainly on so called type 2 inflammation regulated by Th2 lymphocytes and innate lymphoid cells type 2 (ILC2), the role of lung macrophages in promoting allergic reaction is apparent [211]. Macrophages are the most abundant immune cells in the lungs and provide multiple specialized functions based on their anatomical location [211,212].

Traditionally, lung macrophages can be activated via two pathways. The classical pathway of macrophage activation (M1-like) leads to the activation of inflammasomes and is dependent on the Th1 cellular response induced mainly by IFN-γ, TNF-α, and LPS. These macrophages release pro-inflammatory cytokines IL-12, IL-1β, IL-6, and TNF-α [213].

In contrast, lung M2-like macrophages, differentiated by cytokines released from Th2 and ILC2 cells, are preferentially induced in response to the ‘pro-allergic’ cytokines IL-4 and IL-13 [214,215]. These macrophages regulate the expression of genes involved in the removal of dead and dying cells or tissues, participate in anti-inflammatory responses, and mostly secrete IL-10 and TGF-β [216].

By measuring cytokine levels after stimulation of peripheral blood monocytes using LPS as a model of M1 macrophages and by IL-4 to obtain M2 macrophages, IL-4 activated macrophages produced higher levels of IL-6, IL-10, and IL-12p40, but there was no production of IL-12p70 by Th1 cells. Similar observations have been made in bronchoalveolar lavage fluid (BALF) of patients with asthma [217].

Several studies have shown that patients with asthma with allergic airway inflammation have a high number of M2-like macrophages and their products in the BALF and lung tissue [218,219]. Interestingly, recent studies have shown the dynamic nature of lung macrophage subsets during SARS-CoV-2 infection, as observed in animal models utilizing ScRNA-seq of macrophages obtained from BALF. These investigations have revealed significant alterations in the relative proportions of lung macrophage subsets, emphasizing the ability of alveolar macrophages to undergo dynamic changes in response to infection [220].

Macrophages isolated from BALF of patients with allergic asthma have almost three-fold increased levels of MHCII and CD206 compared with that of healthy controls, and also the phenotypic pattern (CCR7^−^CXCR1^+^) suggests their M2a macrophage polarization. Data from patients with bronchial asthma are in agreement with results of experimental studies. Robbe *et al*. studied the M1/M2 polarization of lung macrophages in a mouse model and showed that M1 cells are predominant in non-allergic asthma, whereas the number of M2 cells increase in allergic asthma [221].

Different subsets of M2 macrophages (M2a, M2b, and M2c, M2d) might play diverse roles in different stages of asthma [222,223]. M2a macrophages are predominantly activated by IL-4 and IL-13, which are critical mediators of allergic inflammation. The M2c cells are activated by IL-10 and TGF-β, predominantly produce the anti-inflammatory cytokines TGF-β and IL-10, and play a crucial role in phagocytosis and tissue remodeling, similar to M2b macrophages [222,224]. The secretion of chemokines by individual subsets also differs. CCL17, CCL22, and CCL24 are mainly produced by M2a cells, M2b cells predominantly produce CCL1, while M2c cells produce CCL2 [61,222,225,226].

In childhood asthma, subjects with moderate asthma seem to have more M2c macrophages than children with mild asthma. In addition, the number of M2c cells correlated with IgE levels. The number of M2c cells is higher in asthmatic children requiring hospitalisation during the exacerbation together with decline of M1 population, although there was no difference in the numbers of M2a and M2b cells [227].

Polarisation of the M1/M2 macrophages in asthma can be evaluated also by analysing the expression of NLRP3 inflammasome components, such as nucleotide-binding oligomerization domain-like receptor protein 3. It has been observed that up-regulation of the NLRP3 component nucleotide-binding oligomerization domain-like receptor protein 3 leads to an increase in the levels of IL-4 as well as the release of the pro-inflammatory cytokine IL-1β produced via the caspase-1 pathway. Overexpression of NLRP3 increases IL-4 levels and decreases the M1/M2 ratio, whereas silencing NLRP3 suppresses IL-4 secretion and increases the M1/M2 ratio. NLRP3 deficiency in ovalbumin-induced allergic asthmatic mice increases the number of M1 macrophages and reduces IL-4 levels in BALF. NLRP3 promotes the polarisation of M2 macrophages by up-regulating IL-4 expression. NLRP3, an immune sensor of infection and cellular stress, is associated with the development and exacerbation of asthma.

Regarding evaluation of M2 macrophages in the airways and lungs of patients with bronchial asthma, possible effect of corticosteroids on induction of characteristic M2 markers such as CD163 and CD206 should be taken into account [228–230].

## M1/M2 macrophages in transplantation

The role of macrophages in transplantation is particularly important, as they were shown to play a critical part in graft survival and rejection. In the past, the presence of macrophages in donor organs has been strictly linked to transplant rejection and this process was shown to be mediated by macrophages that have been primed by allogeneic antigens [231–235]. Multiple studies have shown that macrophages play a key role in all phases of allograft rejection; however, their potential in tissue healing and immune tolerance is also undisputable [231,235–244].

While early polarization of macrophages towards an anti-inflammatory M2 state is associated with the induction of immune tolerance, infiltration of the graft with pro-inflammatory M1 macrophages was previously shown to correlate with organ rejection [241,245,246]. Several studies suggest that in transplantation, macrophages shift their polarity in response to various micro-environmental stimuli [247–249]. Furthermore, Zhao et al. demonstrated that the polarization of macrophages into M1 and M2 subsets in mouse heart transplant models was critically dependent on the tumour-necrosis factor receptor associated factor 6 (TRAF6) and the mammalian target of rapamycin (mTOR) proteins, even though the exact mechanism seemed to be rather complex [250].

A recent study by Snyder et al. highlighted, that alveolar macrophages contribute to graft rejection either directly through trained immunity or indirectly via cellular recruitment and other stimulatory effects on donor-specific T cells [251]. Similarly, transcriptional profiling via single-cell RNA sequencing allowed identification of two alveolar macrophage subsets that are uniquely associated with acute lung allograft dysfunction [252]. The authors Moshkelgosha et al. pinpointed specific macrophage populations that participate in lung allograft rejection by recruiting alloreactive T cells. With this in mind, these macrophage subpopulations may indeed represent important therapeutic targets [252].

Classically activated macrophages may predominate in acute rejection, whereas injury-induced macrophages contribute to tissue repair and fibrosis of the graft [236]. Although the macrophage-mediated release of pro-inflammatory cytokines, such as IL-6 and TNF-α following the allorecognition facilitates T-cell responses in transplant rejection [241,253]. Peng et al. demonstrated that macrophages can also produce B-cell activating factor (BAFF), resulting in graft damage during the antibody mediated rejection [243,254].

The role of M2 macrophages in chronic rejection remains a matter of debate with some studies suggesting their protective function, while other studies discuss potentially damaging effects [236].

A recent study investigating diverse aspects of liver transplantation in rats demonstrated that the presence of Kupffer cells with M2 phenotype in liver grafts was associated with better function and healthier structure of liver allografts which ultimately prolonged the recipient’s survival [247]. Across studies, M2 macrophages have been shown to carry anti-inflammatory and graft-protective effects. However, these effects largely depend on the specific transplant context and may vary among different organs.

Further research is currently needed to fully understand the specific roles and mechanisms of M2 macrophages in these complex and diverse environments [236]. While the precise role of M2 macrophages in chronic rejection remains ambiguous, targeting M2 polarization through therapeutic interventions may have a real potential in the treatment of acute rejection. Further research is needed to confirm and clarify these potential mechanisms and effects [236,243,250].

Even though similar cellular mechanisms occur also after haematopoietic stem cell transplantation (HSCT) in a process called graft-versus-host disease (GVHD) the role of macrophages in GVHD is still far from being understood [255]. Clinical trials have shown that patients with a higher M1/M2 ratio have a higher risk of GVHD, and the use of granulocyte colony-stimulating factor (G-CSF) reduces the M1/M2 ratio and thus, significantly decreases the incidence of GVHD [236,256]. Hanaki et al. found that administering donor bone marrow-derived M2 macrophages decreased GVHD severity and improved the overall survival in selected patients [257]. Another retrospective analysis demonstrated that the increased ratio of macrophages to the total nucleated cell number was associated with a risk of delayed engraftment or subsequent graft failure in patients after allogeneic stem cell transplantation [258].

As expected, macrophage subpopulations were postulated to serve as attractive therapeutic targets in solid organ transplantation [259]. Numerous studies suggested that manipulating macrophage activities may bring results in the chronic allograft rejection prevention or treatment [241,243,260,261]. Inhibiting of macrophages by mycophenolate mofetil (MMF) seems to be one but not the only promising strategy [243,260]. A study by Zhang et al. also described regulatory macrophages (Mreg), which represent a subset of macrophages with immunomodulatory properties. As Mregs may be involved in the resolution of various inflammatory conditions, the authors highlight the potential use of Mregs in combination with immunosuppressive agents in various clinical settings. This strategy would involve harnessing the immunomodulatory properties of Mregs to reduce the need for high doses of immunosuppressive agents, thereby minimizing their associated toxicities. However, the relationship between Mregs and transplantation immunity is not fully understood and more studies are needed to elucidate their potential impact on the immune response in transplanted organs [262].

To conclude, the crucial role of macrophages in transplantation is hardly disputable. On one hand, macrophages contribute to the initiation of the inflammatory response leading to the destruction of transplanted tissue. One the other hand, macrophages also play a role in the prevention of organ rejection [231]. However, multiple pathways leading to the production of cytokines, as well as the exact way by which macrophages regulate the tissue repair and healing are far from being fully understood [255,263]. Hence, macrophages continue to remain potential therapeutic target that could help minimize the damage to the transplanted organ and improve the long-term survival of both the organ and organ recipient [243,252,261–264].

## Discussion

**Figure 4:**
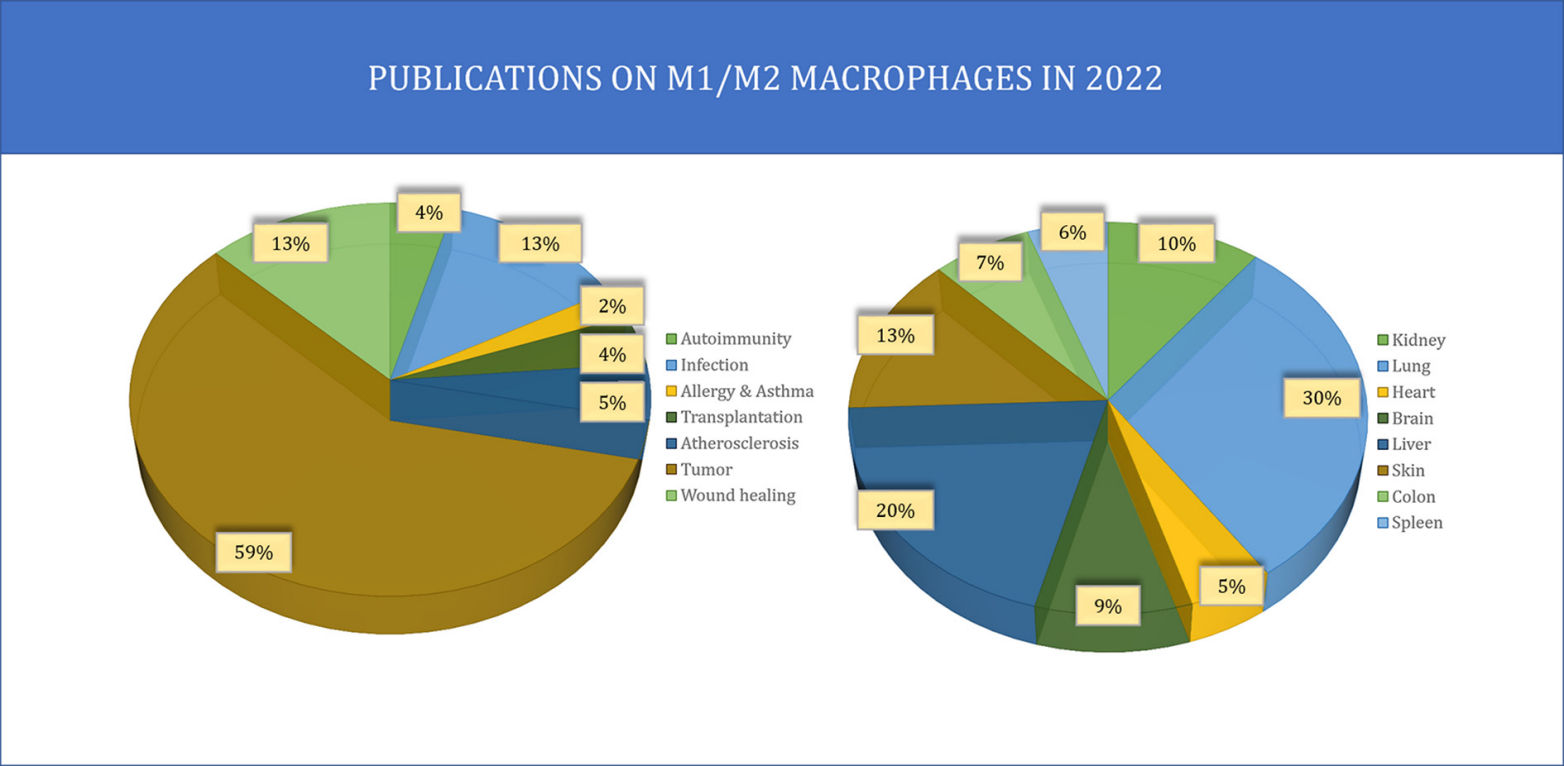
Pie charts illustrating the distribution of research articles on M1/M2 macrophages across different human conditions and organs The left chart displays the number of articles published on M1/M2 macrophages in diverse human conditions. The right chart shows the number of scientific articles published on M1/M2 macrophages in diverse organs. Together, these charts provide insight into the prevalence of research on M1/M2 macrophages in different areas of study and highlight the diverse contexts in which these cells have been investigated.

Macrophages play an important role in the human body. In 2022, the PubMed Central Database reported a total of 1443 publications on the subject of M1/M2 macrophages across various human conditions, along with 791 publications specifically focused on M1/M2 macrophages in specific organs (Figure 4.)

Given their major involvement in the control of inflammation, cancer growth, atherosclerosis, infection, allergy, and transplant rejection, macrophages are polarized into diverse phenotypes according to the changes in their environment. Although the classification of macrophages based on the M1 and M2 phenotypes seems to be rather oversimplified, the extremely heterogenous responses to stimulation highlight the need of a better understanding of macrophage function in human pathologies.

Considering that tissue macrophages from diverse anatomical locations have distinct functional phenotypes, it can be presumed that these diverse functional roles are attributed to the differences in local signals from their tissue of residence (Table 1). Indeed, microenvironmental stimuli contribute to macrophage plasticity and it remains to be answered whether a multiplicity of stimuli could lead to broader heterogeneity in macrophage function.

Macrophages can originate from circulating adult monocytes or from recruited fetal monocytes, as well as from primitive yolk sac-derived macrophages. Under certain conditions and in specific contexts, it has been observed that macrophages can also derive from MDSCs. This phenomenon has been mainly observed in tumors and highlights the plasticity and versatility of MDSCs, which can undergo differentiation into macrophages within specific microenvironments. Although macrophages and microglia are of distinctive origin, in many ways, both cell types exhibit similar specific functions. On the other hand, microglia activation is rather immediate in cases of CNS inflammation, while peripheral macrophages act within days [265]. Thus, we would like to draw attention to the fact that not only microenvironmental stimuli but also the origin and evolution elements affect the biological activity of macrophages giving them properties that are critical for survival.

In human infections, macrophages are essential effectors equipped with a wide variety of antimicrobial and antiviral compounds. To date, the M1 profile appears to be generally protective against bacterial and viral infections. Hence, diverse pathogens learned to engage multiple strategies to evade the antimicrobial and antiviral responses of M1-polarized macrophages. Apart from being capable of inducing very different macrophage phenotypes, bacteria and viruses also induce M2 polarization through modulation of the transcription factors and induction of subsequent changes in protein synthesis. These advanced mechanisms can be observed especially in herpesviruses or intestinal pathogens, such as *S. typhimurium* or *E. coli*. While parasites are known to harm predominantly through the promotion of chronic changes in host’s health and nutritional status, they also secrete immunomodulatory substances that down-regulate parasite-specific effector responses. To date, it is important to understand that among infected individuals, both prolonged M1, as well as M2, macrophage activation is potentially deleterious for the host. While prolonged M2 polarization in HBV infection can accelerate liver fibrosis, prolonged M1 polarization, on the other hand, seems to increase the chances of developing gastric precancerous lesions in *Helicobacter pylori* infection.

Factors driving the recruitment and functional skewing of macrophages in the TME have gained particular interest in the last decade. The infiltration with the M2 phenotype has been shown to serve as a reliable prognostic marker carrying an exceptional repertoire of tumor-promoting capabilities. While both M1 and M2 macrophages are involved in the intratumoural inflammation, M2 macrophages are key modulators of immunosuppression, angiogenesis, and neovascularization, impacting negatively patients’ prognoses. However, under specific conditions M1 macrophages can also exhibit pro-tumorigenic properties and this paradoxical effect of M1 macrophages highlights the complexity of immune response to cancer. Despite the varying diversity and plasticity of macrophages observed under different pathologic conditions, it seems that the M1/M2 classification does reflect the opposing properties of macrophage subsets in tumours. For that reason, a number of macrophage-centered therapeutic anti-tumoural strategies are being under investigation.

The redundancy of regulatory pathways leading to the activation of macrophages in inflammatory conditions raises many questions regarding the role of macrophage polarization in autoimmune diseases. Both prolonged activation of M1 macrophages, as well as the altered function of M2 macrophages, can initiate and further control the breakdown of immunological tolerance leading to autoimmunity. Furthermore, the disease activity reflected by the periods of remission and disease flares is driven also by the M1/M2 imbalance, especially in SLE and RA, suggesting a potential therapeutic role of macrophages in autoimmunity.

Vascular macrophages play a central role in the development of plaques in atherosclerotic cardiovascular disease. Interestingly, both M1 and M2 macrophage profiles were previously associated with the progression of atherosclerotic plaques by affecting cholesterol metabolism and modulating matrix metalloproteinases/extracellular matrix ratio. While M1 macrophages seem to affect plaque stability and thus, promote rupturing of the plaques, M2 macrophages were shown to contribute to plaque calcification, higher necrotic content, and increased size. Hence, the M1 and M2 classification does not signify opposing roles in atherosclerosis and rather indicates a synergic role of phenotypically and functionally different macrophage subtypes.

In the lungs, macrophages represent the most abundant cell type and their activation pathways are dependent on the intercellular interactions and communication. Plasticity of lung macrophages has been heavily associated with the development of asthma. In general, M2 macrophages are critical mediators of allergic inflammation and diverse M2 subsets are involved in different stages of asthma. As it has been already shown, the role of macrophages largely depends on the cytokine profile with Th2 cytokines skewing macrophage differentiation to M2 type. In addition, alveolar macrophages also contribute to the graft rejection in lung transplantation.

Solid organ transplantation is a therapeutic option for patients with end-stage organ failure. Understanding the mechanism behind organ rejection is one of the greatest challenges in transplant medicine. Macrophages seem to have dual role in transplant rejection, where classically activated M1 macrophages were shown to predominate in acute rejection, whereas upon the response to different cytokine profiles, M2 macrophages contribute to tissue repair and fibrosis of the graft. The involvement of macrophages in transplant rejection is very complex as macrophages can act both directly through trained immunity, as well as indirectly via cellular recruitment and other stimulatory effects on donor-specific T cells. The M1/M2 classification remains a matter of debate in organ transplantation. While pro-inflammatory M1 profile seems to correlate with acute organ rejection, the exact role of M2 macrophages in chronic rejection is still poorly understood with many studies presenting opposing conclusions ranging from the protective to detrimental role of M2 macrophages in graft loss.

In conclusion, macrophages are immune cells with high phenotypic heterogeneity that respond to various stimuli leading to the activation of distinct signaling pathways. Although, the M1/M2 classification is generally oversimplified, under specific immunopathologic conditions, such as the tumor-immune system interplay or the host–pathogen interaction during microbial pathogenesis, M1 and M2 macrophages were shown to harness contrasting signalizations and under these circumstances, the opposing activities indeed follow the macrophage M1/M2 phenotypes paradigm. Our understanding of the latest research leads us to conclude that it would be more precise to describe macrophages as M1-like and M2-like, rather than to use the conventional M1 and M2 nomenclature.

## Data Availability

The manuscript does not contain any original data.

## Abbreviations

BALF: bronchoalveolar lavage fluid
CCL: chemokine (C-C motif) ligand
EGF: epidermal growth factor
ESAT-6: early secreted antigenic target 6kDa protein
GM-CSF: granulocyte-macrophage colony-stimulating factor
GvHD: graft-versus-host disease
HBV: hepatitis B virus
HSCT: haematopoietic stem cell transplantation
IFN-γ: interferon gamma
IL: interleukin
ILC2: innate lymphoid cells type 2
iNOS: inducible nitric oxide synthase
LPS: lipopolysaccharide
M-CSF: macrophage colony-stimulating factor
MHC: major histocompatibility complex
mTOR: mammalian target of rapamycin
NFκB: nuclear factor kappa-B
NLRP3: NOD-like receptor family, pyrin domain containing 3
NO: nitric oxide
PD-L1: programmed cell death ligand 1
PGE2: prostaglandin E2
RA: rheumatoid arthritis
RANKL: receptor activator of nuclear factor kappa B ligand
RNS: reactive nitrogen species
ROS: reactive oxygen species
SLE: systemic lupus erythematosus
TAMs: tumor-associated macrophages
TGF: transforming growth factor
TIM-3: T cell immunoglobulin and mucin-domain containing 3
TLR: Toll-like receptor
TME: tumor microenvironment
TNF: tumor necrosis factor
TRAF6: tumour-necrosis factor receptor associated factor 6
VEGF: vascular endothelial growth factor
